# Incidence of venous thromboembolism after surgery for adenocarcinoma *in situ* and the validity of the modified Caprini score: A propensity score-matched study

**DOI:** 10.3389/fonc.2022.976988

**Published:** 2022-09-02

**Authors:** Yong-sheng Cai, Hong-hong Dong, Xin-yang Li, Xin Ye, Shuo Chen, Bin Hu, Hui Li, Jin-bai Miao, Qi-rui Chen

**Affiliations:** Department of Thoracic Surgery, Beijing Institute of Respiratory Medicine and Beijing Chao-Yang Hospital, Capital Medical University, Beijing, China

**Keywords:** adenocarcinoma *in situ* (AIS), venous thromboembolism (VTE), propensity score matching (PSM), modified caprini risk assessment model (Caprini RAM), world health organization (WHO)

## Abstract

**Background:**

Recently, the new World Health Organization (WHO) tumor classification removed adenocarcinoma *in situ* (AIS) from the diagnosis of lung cancer. However, it remains unclear whether the “malignancy” item should be assessed when the modified Caprini Risk Assessment Model (RAM) is used to assess venous thromboembolism (VTE) risk in AIS. The purpose of our study is to assess differences between AIS and stage IA adenocarcinoma (AD) from a VTE perspective.

**Methods:**

A retrospective study was performed on AIS and IA adenocarcinoma in our hospital from January 2018 to December 2021, and divided into AIS group and AD group. Propensity score matching (PSM) was used to compare the incidence of VTE and coagulation function, and to analyze whether the RAM is more effective when the “malignancy” item is not evaluated in AIS.

**Results:**

491 patients were included after screening, including 104 patients in the AIS group and 387 patients in the AD group. After PSM, 83 patients were matched. The incidence of VTE and D-dimer in the AIS group was significantly lower than that in the AD group (P<0.05).When using the RAM to score AIS, compared with retaining the “malignancy” item, the incidence of VTE in the intermediate-high-risk group was significantly higher after removing the item (7.9% vs. 36.4%, P=0.018), which significantly improved the stratification effect of the model.

**Conclusions:**

The incidence of postoperative VTE in AIS was significantly lower than that in stage IA adenocarcinoma. The stratification effect was more favorable when the “malignancy” item was not evaluated in AIS using the RAM.

## Introduction

Venous thromboembolism (VTE) is a common complication in patients with malignant tumors, mainly including deep vein thrombosis (DVT) and pulmonary embolism (PE). Once it occurs, it significantly increases the patient’s risk of postoperative complications and death (1, 2). The risk of VTE in cancer patients is significantly higher than that in the general population, especially lung cancer, which is the malignant tumor with the highest incidence of VTE (3, 4). This was confirmed in our previous study, with a 15% incidence of VTE after lung cancer surgery (5). Therefore, lung cancer patients undergoing surgical treatment are a high-risk group for VTE.

To better identify patients at high risk of VTE, a variety of VTE risk assessment models have been developed, among which the modified Caprini RAM is widely used in thoracic surgery (6). High-risk factors for VTE include patient-related risk factors (age, body mass index, abnormal lung function, varicose veins, confined to bed, etc.), tumor-related risk factors (current malignant tumor, history of tumor, etc.), and treatment-related factors (operation time, central venous access, chemotherapy, etc.). In addition, the guidelines recommend perioperative VTE prophylaxis in patients after surgery at intermediate and high risk as assessed by the use of the modified Caprini RAM (7, 8). In the current real world, with the improvement of health awareness and the promotion of low-dose chest CT in lung cancer screening, pulmonary nodules are becoming increasingly common in the clinical work of thoracic surgery, especially ground glass opacities (GGOs) (9–11). Among them, the number of patients with the postoperative pathology of AIS is also increasing.

At present, the tumor classification of the World Health Organization (WHO) is regarded as an internationally recognized tumor diagnostic standard, which is crucial for guiding clinical treatment. Compared with the fourth edition of the WHO classification released in May 2015 (12), the fifth edition of the WHO classification released in April 2021 rearranged lung tumors and removed AIS from the list of adenocarcinomas (13). AIS is no longer classified as a malignant tumor and is classified as a precursor gland lesion. Whether the “malignancy” item should be evaluated in patients with AIS using the modified Caprini RAM is unclear. Therefore, this study aimed to compare the incidence of VTE between patients with AIS and stage IA adenocarcinoma by propensity score matching (PSM) and to evaluate the effectiveness of the modified Caprini RAM in predicting postoperative VTE in patients with AIS.

## Materials and methods

### Patient selection

**Figure 1:**
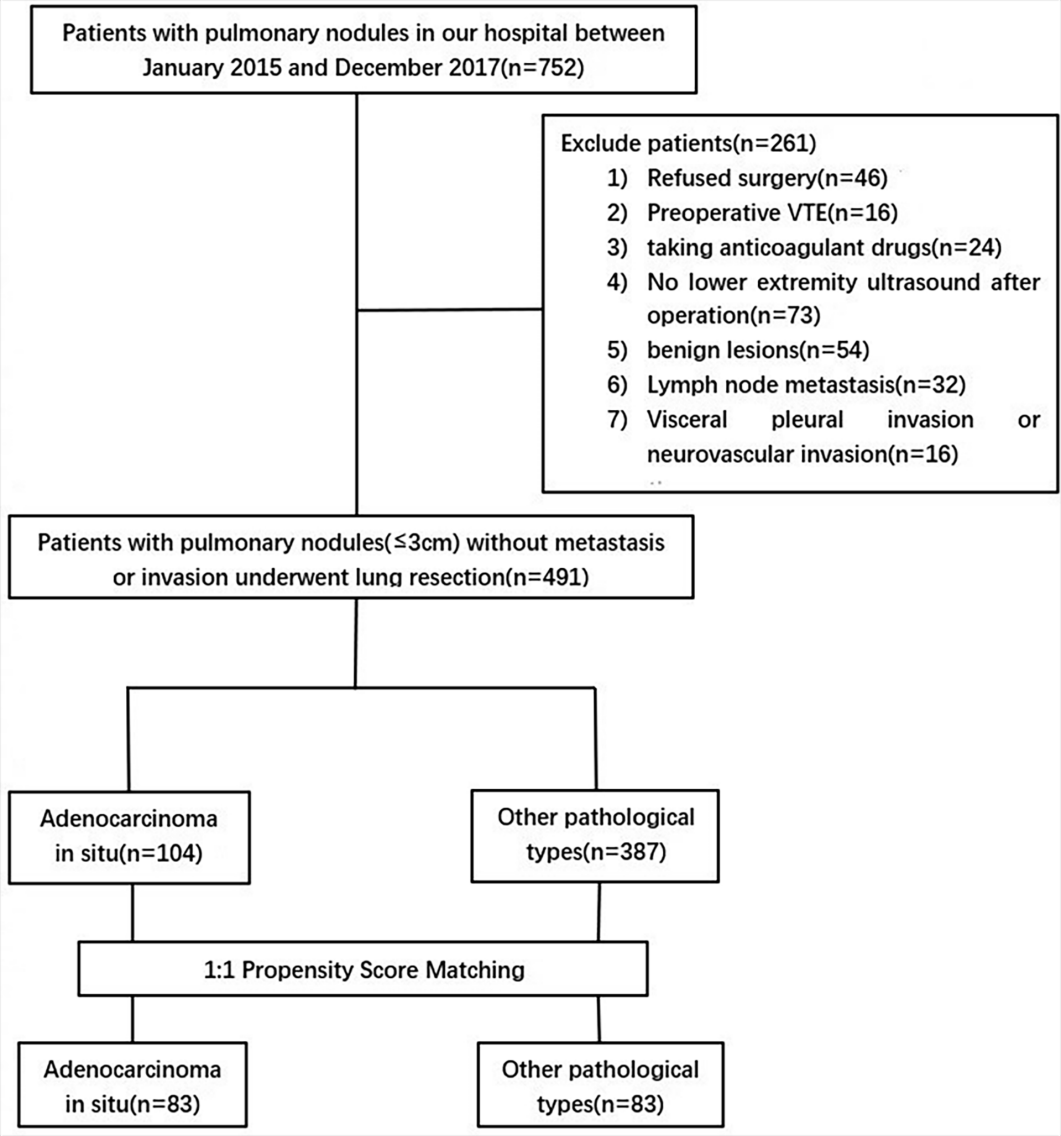
Flowchart showing the patient selection criteria. VTE,venous thromboembolism.

This study is a single-center retrospective study that was approved by the Ethics Committee of Beijing Chaoyang Hospital Affiliated to Capital Medical University (2017-Ke-1), and patients were exempted from informed consent. The clinical data of 752 patients with clinical stage IA disease from January 2018 to December 2021 were retrospectively collected. Clinical and pathological TNM staging was determined according to the eighth edition of lung cancer staging published by the American Joint Committee on Cancer/International Union Against Cancer (14). The inclusion criteria were as follows: (1) postoperative pathological diagnosis of primary AIS or stage IA adenocarcinoma; (2) lower extremity venous ultrasound before and after surgery; and (3) preoperative lower extremity venous ultrasound showed no DVT. The exclusion criteria were as follows: (1) postoperative pathological diagnosis of benign lesions; (2) pathological diameter greater than 3 cm, lymph node metastasis or invasion of pleura, neurovascular, etc.; (3) preoperative lower extremity ultrasonography diagnosed as DVT; (4) no lower extremity venous ultrasound was performed postoperatively; (5) anticoagulant drugs were required for any reason during the perioperative period; and (6) the patient refused surgical treatment. Finally, a total of 491 patients were included in this study (**Figure 1**). The follow-up period ended at discharge.

### Data collection

The following clinical data were collected through the electronic medical record system: age, sex, body mass index (BMI), hospital stay, acute myocardial infarction (<1 month), congestive heart failure (<1 month), history of inflammatory bowel disease, history of prior major surgery (<1 month), complications of pregnancy, oral contraceptives or hormone replacement therapy (HRT), sepsis (<1 month), severe acute lung disease (<1 month), comorbidities (hypertension, diabetes, coronary heart disease, hyperlipidemia), smoking history, drinking history, family history, history of malignant tumor, previous history of VTE, confined to bed >72 hours, central venous access, history of chemotherapy, abnormal pulmonary function, swollen legs (current), varicose veins, intermuscular vein dilation, positive anticardiolipin antibody, positive lupus anticoagulant, acute spinal cord injury (<1 month), operation-related information (surgical approach, resection range, operation time, blood loss, and number of lymph nodes removed), pathological diameter, tumor location, nodule morphology, forced expiratory volume in one second (FEV1), forced vital capacity (FVC), maximal voluntary ventilation (MVV), platelets (PLT), prothrombin time (PT), partial thromboplastin time (APTT), D-dimer, and VTE events.

In this study, all enrolled patients underwent preoperative and postoperative lower extremity venous ultrasonography to evaluate the presence or absence of DVT. If the patient had typical symptoms of PE (chest pain, hemoptysis, dyspnea or persistent unexplained hypoxemia), a modified Caprini score ≥9, or newly diagnosed DVT after surgery, computed tomography pulmonary angiography (CTPA) was performed.

### Evaluation of VTE and coagulation

A VTE event was defined as postoperative VTE in patients with no preoperative diagnosis of VTE. The evaluation of coagulation indicators included preoperative and postoperative PLT, PT, APTT, and D-dimer. The preoperative coagulation results of all patients were blood drawn and submitted on the first day of hospitalization; the postoperative coagulation results were blood drawn and submitted on the first postoperative day. The PSM method was used to evaluate the differences in the incidence of VTE and coagulation between the two groups of patients with AIS and stage IA adenocarcinoma.

### Evaluation of the modified Caprini RAM

The modified Caprini RAM was used to assess the risk of VTE according to clinical parameters (**Supplementary Table**). In this study, all clinical parameters in the model were collected for PSM, and the incidence of VTE in patients with AIS and stage IA adenocarcinoma was compared.

All patients underwent modified Caprini RAM risk stratification, with ≤4 scores indicating low risk, 5-8 scores indicating risk, and ≥9 scores indicating high risk. In this study, patients with AIS were divided into two groups according to the score with keeping and removing the “malignant tumor” item, and the proportion of the intermediate-high-risk population in the two groups was compared to evaluate the effectiveness of the modified Caprini RAM for AIS patients.

### Statistical analysis

Continuous variables with a normal distribution are expressed as the mean ± standard deviation (mean ± SD), and a t-test was used for comparisons between groups. Continuous variables that did not conform to a normal distribution are represented by M (Q1, Q3), and comparisons between groups were performed using the Mann–Whitney U test. Categorical variables are represented by percentages using the χ2 test or Fisher’s exact test. To eliminate the effects of confounding factors and selection bias, 1:1 PSM was used to match the two groups of patients (PS = 0.02). Matched variables included clinical parameters in the modified Caprini RAM scale, comorbidities (hypertension, diabetes, coronary heart disease, and hyperlipidemia), smoking history, drinking history, family history, intermuscular vein dilatation, surgery-related information (surgical approach, resection range, operation time, blood loss, and number of lymph nodes removed), pathological diameter, tumor location, nodule morphology, FEV1, FVC, and MVV. Outcome variables were the above coagulation indicators and VTE events. All analyses in this study were performed using SPSS version 26.0 software (IBM, Armonk, NY, USA), and a two-sided P value of <0.05 was considered statistically significant.

## Results

### Baseline characteristics

Among the 491 patients enrolled, 104 patients (21%) were diagnosed with AIS postoperatively, and 387 patients (79%) were diagnosed with stage IA adenocarcinoma. The baseline data of all patients are shown in **Table 1**. There were significant differences in age, sex and length of hospital stay between the two groups (P<0.05). Compared with the AD group, the AIS group had a higher proportion of females, younger age, and shorter hospital stay. However, there was no significant difference in BMI or comorbidities (hypertension, diabetes, CHD, and hyperlipidemia) between the two groups (P>0.05). Comparing the past histories of the two groups, the results showed that the proportions of smoking and drinking history in the AD group were significantly higher than those in the AIS group (20.2% vs. 9.7%, 10.9% vs. 2.9%, P<0.05), but in acute myocardial infarction (<1 month), congestive heart failure (<1 month), inflammatory bowel disease, previous major surgery (<1 month), complications of pregnancy, oral contraceptives or HRT, sepsis (<1 month), severe acute lung disease (<1 month), tumor history, family history, history of VTE, and acute spinal cord injury (<1 month) the results were not significantly different (P>0.05). There were significant differences in abnormal pulmonary function, MVV, and varicose veins between the two groups (P<0.05). The AD group had significantly more patients with abnormal lung function (13.4% vs. 3.8%, P=0.006) and varicose veins (3.6% vs. 0%, P=0.049) than the AIS group, and the MVV in the AD group was significantly lower than that in the AIS group (107.28 ± 29.26 vs. 113.9 ± 30.2, P=0.042); however, there were no significant differences in FEV1, FVC, confined to bed >72 hours, central venous access, history of chemotherapy, intermuscular vein dilation, positive anticardiolipin antibody, and positive lupus anticoagulant in both groups. The surgical and pathological information of the two groups was compared. The results showed that the pathological diameter (0.79 ± 0.31 vs. 1.35 ± 0.61, P<0.001), the number of lymph nodes removed (4.82 ± 4.51 vs. 11.32 ± 7.81, P<0.001) and blood loss in the AIS group (51.97 ± 65.02 vs. 87.01 ± 148.39, P<0.001) were significantly lower than those in the AD group, and there were also significant differences in nodule morphology and resection range between the two groups. The nodules in the AIS group were mainly ground glass opacities (GGOs) (79.8%) upon imaging, and the resection range was mostly sublobar resection (83.7%). However, the operation approach, operation time and tumor location were all comparable (P>0.05).

**Table 1:** Comparison of baseline data between AIS group and AD group before PSM.

Variable	AIS (n=104)	N-AIS (n=387)	*Z/t/X²*	*P-Value*
Gender^a^			5.398	0.020
male	26 (25%)	144 (37.2%)		
female	78 (75%)	243 (62.8%)		
Age (years)^a^			/	0.001
<40	20 (19.2%)	26 (6.7%)		
40-59	50 (48.1%)	189 (48.8%)		
60-74	31 (29.8%)	163 (42.1%)		
≥75	3 (2.9%)	9 (2.3%)		
BMI≥30 (kg/m²)^a^	5 (4.8%)	20 (5.2%)	0.022	0.882
Hospital stay (d)^b^	7.45 ± 2.62	9.96 ± 4.66	-7.190	<0.001
Acute myocardial infarction (<1mo)^a^	0 (0%)	0 (0%)	/	1.000
Congestive heart failure (<1 mo)^a^	0 (0%)	0 (0%)	/	1.000
History of inflammatory bowel disease^a^	0 (0%)	0 (0%)	/	1.000
History of prior major surgery (<1 mo)^a^	0 (0%)	0 (0%)	/	1.000
Complications of pregnancy^a^	0 (0%)	0 (0%)	/	1.000
Oral contraceptive use or HRT^a^	0 (0%)	0 (0%)	/	1.000
Sepsis (<1 mo)^a^	0 (0%)	0 (0%)	/	1.000
serious acute lung disease (<1 mo)^a^	0 (0%)	0 (0%)	/	1.000
Hypertension^a^	29 (27.9%)	114 (29.5%)	0.098	0.754
Diabetes^a^	7 (6.7%)	37 (9.6%)	0.805	0.370
CHD^a^	2 (1.9%)	23 (5.9%)	2.741	0.098
Hyperlipidemia^a^	10 (9.7%)	24 (6.2%)	1.482	0.223
History of tumor^a^	2 (1.9%)	16 (4.1%)	/	0.387
Smoking history^a^	10 (9.7%)	78 (20.2%)	6.190	0.013
Drinking history^a^	3 (2.9%)	42 (10.9%)	6.252	0.012
Family history^a^	8 (7.7%)	36 (9.3%)	0.260	0.610
History of VTE^a^	0 (0.0%)	2 (0.5%)	/	1.000
Family history of VTE^a^	0 (0%)	0 (0%)	/	1.000
Confined to bed (>72 h)^a^	1 (1.0%)	7 (1.8%)	/	1.000
Central venous access^a^	0 (0%)	4 (1.0%)	/	0.583
Chemotherapy^a^	2 (1.9%)	8 (2.1%)	/	1.000
Abnormal pulmonary function^a^	4 (3.8%)	52 (13.4%)	7.462	0.006
swollen legs (current)^a^	0 (0%)	0 (0%)	/	1.000
Varicose veins^a^	0 (0.0%)	14 (3.6%)	3.873	0.049
Intermuscular vein dilation^a^	11 (10.6%)	56 (14.5%)	1.054	0.304
Surgical approach^a^			/	0.589
VATS	104 (100%)	382 (98.7%)		
Open	0 (0%)	5 (1.3%)		
Resection range^a^			101.81	<0.001
Sublobectomy	87 (83.7%)	112 (28.9%)		
Lobectomy	17 (16.3%)	275 (71.1%)		
Operation time^a^			4.303	0.116
<45min	4 (3.8%)	4 (1.0%)		
45-360min	100 (96.2%)	382 (98.7%)		
≥360min	0 (0%)	1 (0.3%)		
Positive anticardiolipin antibody^a^	0 (0%)	0 (0%)	/	1.000
Positive Lupus anticoagulant^a^	0 (0%)	0 (0%)	/	1.000
Acute spinal cord injury (<1 mo)^a^	0 (0%)	0 (0%)	/	1.000
Blood loss^b^	51.97 ± 65.02	87.01 ± 148.39	-3.548	<0.001
Number of LNR^b^	4.82 ± 4.51	11.32 ± 7.81	-10.94	<0.001
Pathological diameter^b^	0.79 ± 0.31	1.35 ± 0.61	-12.86	<0.001
Tumor location^a^			5.426	0.246
LU	33 (31.7%)	95 (24.5%)		
LL	19 (18.3%)	54 (14%)		
RU	35 (19.8%)	142 (36.7%)		
RM	5 (4.8%)	30 (7.8%)		
RL	12 (11.5%)	66 (17.1%)		
Nodule morphology^a^			66.182	<0.001
GGO	88 (79.8%)	138 (35.7%)		
Subsolid	16 (15.4%)	135 (34.9%)		
Solid	5 (4.8%)	114 (29.4%)		
FEV1^b^	2.73 ± 0.72	2.61 ± 0.65	1.587	0.113
FVC^b^	3.44 ± 0.87	3.41 ± 0.79	0.277	0.782
MVV^b^	113.9 ± 30.20	107.28 ± 29.26	2.034	0.042

AIS, adenocarcinoma in situ; AD, adenocarcinoma; PSM, propensity score matching; BMI, body mass index; HRT, hormone replacement therapy; CHD, coronary heart disease; VTE, venous thromboembolism; VATS, video-assisted  thoracic surgery; LNR, lymph node removal; LU, left upper lobe; LL, left lower lobe; RU, right upper lobe; RM, right middle lobe; RL, right lower lobe; GGO, ground glass opacity; FEV1, forced expiratory volume in one second; FVC, forced vital capacity; MVV, maximal voluntary ventilation.^a^:n(%); ^b^:mean ± SD; SD, standard deviation.

### Propensity score matching

**Figure 2:**
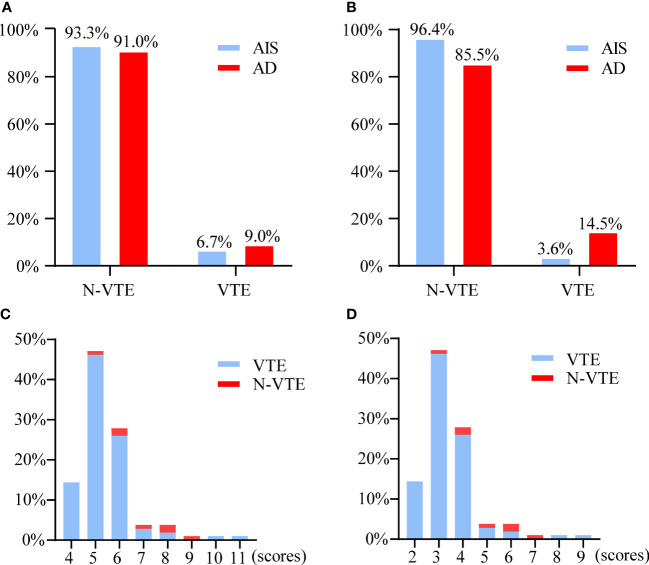
**(A)** Proportion of VTE and N-VTE patients between AIS and AD groups before PSM. **(B)** Proportion of VTE and N-VTE patients between AIS and AD groups after PSM. **(C)** Distribution of Caprini scores in patients with AIS while remaining the “malignant tumor” item. **(D)** Distribution of Caprini scores in patients with AIS while removing the “malignant tumor” item. VTE, venous thromboembolism. AIS, adenocarcinoma in situ.PSM, propensity score matching. AD, adenocarcinoma.

Before PSM, there were significant differences in age, sex, length of hospital stay, smoking history, drinking history, abnormal lung function, MVV, varicose veins, pathological diameter, number of lymph nodes removed, blood loss, nodule morphology, and resection range between the two groups of patients (**Table 1**). The incidence of VTE and coagulation indicators were compared between the two groups. The results showed that (**Figure 2A**) there were 7 and 35 patients with VTE events in the AIS group and AD group, respectively, and there was no significant difference in the incidence of VTE between the two groups (6.7% vs. 9.0%, P>0.05). There was also no significant difference in coagulation indices preoperatively and postoperatively between the two groups (**Table 2**).

**Table 2:** Comparison of coagulation between AIS group and AD group before and after PSM.

PSM	Variable	AIS (n=104)	N-AIS (n=387)	*Z/t/X²*	*P*-Value
Before PSM	Pre-PLT	218.78 ± 74.70	225.78 ± 60.73	-1.070	0.285
	Pre-APTT	25.65 ± 4.34	25.93 ± 5.80	-0.456	0.649
	Pre-PT	11.48 ± 1.73	11.71 ± 0.65	-1.283	0.202
	Pre-D-Dimer	0.29 ± 0.36	0.32 ± 0.44	-0.587	0.557
	Post-PLT	220.33 ± 52.36	213.50 ± 53.43	1.161	0.246
	Post-APTT	25.33 ± 2.18	25.35 ± 3.10	-0.076	0.940
	Post-PT	12.11 ± 0.68	12.12 ± 0.70	-0.158	0.875
	Post-D-Dimer	1.15 ± 1.02	1.42 ± 2.08	-1.323	0.186
After PSM	Pre-PLT	219.03 ± 73.72	228.11 ± 72.18	-0.800	0.425
	Pre-APTT	25.49 ± 4.68	26.02 ± 2.53	-0.902	0.368
	Pre-PT	11.42 ± 1.91	11.68 ± 0.64	-1.192	0.235
	Pre-D-Dimer	0.29 ± 0.39	0.51 ± 0.92	-2.001	**0.048**
	Post-PLT	222.00 ± 52.09	218.77 ± 49.74	0.408	0.684
	Post-APTT	25.41 ± 2.27	25.25 ± 2.02	0.469	0.640
	Post-PT	12.08 ± 0.64	12.10 ± 0.66	-0.229	0.820
	Post-D-Dimer	1.11 ± 0.94	1.77 ± 2.73	-2.078	**0.040**

AIS, adenocarcinoma in situ; AD, adenocarcinoma; PSM, propensity score matching; Pre-, preoperative; Post-, postoperative; PLT, platelet; APTT, activated partial thromboplastin time; PT, Prothrombin time. Values in bold are both less than 0.05, indicating a statistically significant difference.

After PSM, the baseline data of 83 patients were re-evaluated. The results are shown in **Table 3**. There was no significant difference in any baseline variable between the two groups (P>0.05). Re-evaluation of the incidence of VTE and coagulation indicators showed that (**Figure 2B**) there were 3 and 12 patients with VTE events in the AIS group and AD group, respectively, and there was a significant difference in the incidence of VTE between the two groups (3.6% vs. 14.5%, P =0.015). In terms of coagulation indicators (**Table 2**), the preoperative and postoperative D-dimer levels in the AIS group were significantly lower than those in the AD group (0.29 ± 0.39 vs. 0.51 ± 0.92; 1.11 ± 0.94 vs. 1.77 ± 2.73, P<0.05).

**Table 3:** Comparison of baseline data between AIS group and AD group after PSM.

Variable	AIS (n=83)	N-AIS (n=83)	*t/X²*	*P-Value*
Gender^a^			0.495	0.482
male	24 (28.9%)	20 (24.1%)		
female	59 (71.4%)	63 (75.9%)		
Age (years)^a^			/	0.497
<40	17 (20.5%)	14 (16.9%)		
40-59	40 (48.2%)	35 (42.2%)		
60-74	24 (28.9%)	29 (34.9%)		
≥75	2 (2.4%)	5 (6.0%)		
BMI≥30 (kg/m²)^a^	3 (3.6%)	4 (4.8%)	/	1.000
Hospital stay (d)^b^	7.63 ± 2.717	7.84 ± 3.121	-0.477	0.634
Acute myocardial infarction (<1mo)^a^	0 (0%)	0 (0%)	/	1.000
Congestive heart failure (<1 mo)^a^	0 (0%)	0 (0%)	/	1.000
History of inflammatory bowel disease^a^	0 (0%)	0 (0%)	/	1.000
History of prior major surgery (<1 mo)^a^	0 (0%)	0 (0%)	/	1.000
Complications of pregnancy^a^	0 (0%)	0 (0%)	/	1.000
Oral contraceptive use or HRT^a^	0 (0%)	0 (0%)	/	1.000
Sepsis (<1 mo)^a^	0 (0%)	0 (0%)	/	1.000
serious acute lung disease (<1 mo)^a^	0 (0%)	0 (0%)	/	1.000
Hypertension^a^	21 (25.3%)	29 (34.9%)	1.832	0.176
Diabetes^a^	5 (6.0%)	8 (9.6%)	0.751	0.386
CHD^a^	2 (2.4%)	3 (3.6%)	/	1.000
Hyperlipidemia^a^	7 (8.4%)	9 (10.8%)	0.277	0.599
History of tumor^a^	1 (1.2%)	4 (4.8%)	/	0.361
Smoking history^a^	9 (10.8%)	10 (12%)	0.059	0.807
Drinking history^a^	8 (9.6%)	8 (9.6%)	/	1.000
Family history^a^	5 (6.0%)	4 (4.8%)	/	1.000
History of VTE^a^	0 (0%)	0 (0%)	/	1.000
Family history of VTE^a^	0 (0%)	0 (0%)	/	1.000
Confined to bed (>72 h)^a^	1 (1.2%)	1 (1.2%)	/	1.000
Central venous access^a^	0 (0%)	0 (0%)	/	1.000
Chemotherapy^a^	1 (1.2%)	4 (4.8)	/	0.367
Abnormal pulmonary function^a^	4 (4.8%)	5 (6.0%)	/	1.000
swollen legs (current)^a^	0 (0%)	0 (0%)	/	1.000
Varicose veins^a^	0 (0%)	0 (0%)	/	1.000
Intermuscular vein dilation^a^	8 (9.6%)	14 (16.9%)	1.886	0.170
Surgical approach^a^			/	1.000
VATS	83 (100%)	83 (100%)		
Open	0 (0%)	0 (0%)		
Resection range^a^			2.184	0.139
Sublobectomy	68 (81.9%)	60 (72.3%)		
Lobectomy	15 (18.1%)	23 (27.7%)		
Operation time^a^			/	1.000
<45min	2 (2.4%)	3 (3.6%)		
45-360min	81 (97.6%)	80 (96.4%)		
≥360min	0 (0%)	0 (0%)		
Positive anticardiolipin antibody^a^	0 (0%)	0 (0%)	/	1.000
Positive Lupus anticoagulant^a^	0 (0%)	0 (0%)	/	1.000
Acute spinal cord injury (<1 mo)^a^	0 (0%)	0 (0%)	/	1.000
Blood loss^b^	56.75 ± 71.59	56.69 ± 66.99	0.006	0.996
Number of LNR^b^	5.37 ± 4.75	5.20 ± 5.75	0.206	0.837
Pathological diameter^b^	0.82 ± 0.32	0.85 ± 0.32	-0.464	0.643
Tumor location^a^			2.83	0.587
LU	28 (33.7%)	25 (30.1%)		
LL	14 (16.9%)	10 (12.0%)		
RU	28 (33.7%)	33 (39.8%)		
RM	4 (4.8%)	8 (9.6%)		
RL	9 (10.8%)	7 (8.4%)		
Nodule morphology^a^			0.633	0.729
GGO	64 (77.1%)	64 (77.1%)		
Subsolid	14 (16.9%)	16 (19.3%)		
Solid	5 (6.0%)	3 (3.6%)		
FEV1^b^	2.75 ± 0.76	2.68 ± 0.67	0.65	0.517
FVC^b^	3.47 ± 0.90	3.41 ± 0.76	0.478	0.633
MVV^b^	114.64 ± 30.82	107.87 ± 30.01	1.425	0.156

AIS, adenocarcinoma in situ; AD, adenocarcinoma; VTE, PSM, propensity score matching; BMI, body mass index; HRT, hormone replacement therapy; CHD, coronary heart disease; VTE, venous thromboembolism; VATS, video-assisted  thoracic surgery; LNR, lymph node removal; LU, left upper lobe; LL, left lower lobe; RU, right upper lobe; RM, right middle lobe; RL, right lower lobe; GGO, ground glass opacity; FEV1, forced expiratory volume in one second; FVC, forced vital capacity; MVV, maximal voluntary ventilation.^a^:n (%); ^b^:mean ± SD; SD, standard deviation.

### Effect of the modified Caprini RAM

**Figure 3:**
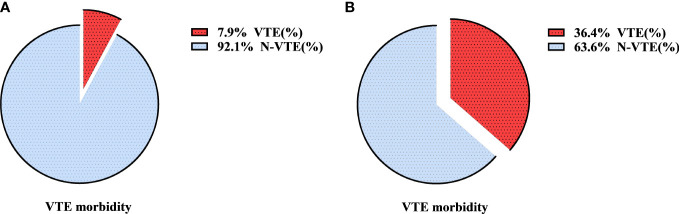
The incidence of VTE in intermediate-high risk with AIS after modified Caprini RAM when remaining the “malignant tumor” item **(A)** or removing the “malignant tumor” item **(B)**. VTE, venous thromboembolism; RAM, risk assessment model; AIS, adenocarcinoma *in situ*.

All patients with AIS (n=104) were assigned a risk score according to the modified Caprini RAM, in which “malignant tumor” accounted for 2 scores. When the “malignant tumor” item score was retained, the distributions of the Caprini score and VTE are shown in **Figure 2C**. There were 15, 86, and 3 patients in the low-, intermediate-, and high-risk groups, respectively. In addition, 0, 6, and 1 patients in the three groups had VTE events, mainly in the intermediate-risk group (5-8 scores). When the “malignant tumor” item was not evaluated, the distributions of the Caprini score and VTE are shown in **Figure 2D**. There were 93, 10, and 1 patients in the low-, intermediate-, and high-risk groups, respectively. Among the three groups, VTE events occurred in 3, 4, and 0 patients, respectively. The incidence of VTE in the intermediate-high-risk patients was compared between the above two scores. The results are shown in **Figure 3**. The incidence of VTE was 7.9% and 36.4%, respectively, with a significant difference (P=0.018). For AIS, the modified Caprini RAM was more effective in predicting postoperative VTE in intermediate-high-risk patients when the “malignancy” item was not evaluated.

## Discussion

At present, the incidence of postoperative VTE in patients with AIS is unclear. This study confirmed that the overall incidence of postoperative VTE was 6.7%, which is similar to the incidence of VTE after lung resection for benign lesions (7%) in our previous study (5). Many previous studies have confirmed that AIS mostly appears as pure ground-glass opacities (p-GGOs) on imaging (15–17). In recent years, the widespread promotion and application of low-dose chest computed tomography (CT) has facilitated the detection of pulmonary nodules in many asymptomatic individuals. Among them, isolated ground-glass opacities (GGOs) have become an emerging group in thoracic surgery (18). This study showed that 79.8% of AIS patients showed p-GGOs on CT, which was consistent with the results of previous studies (15, 16). Ishida et al. (15) reported that 74% of AIS patients showed p-GGOs on CT, and Jia et al. (16) also reported that 78% of AIS patients showed p-GGOs on CT. It can be seen that with the increase in the detection rate of GGOs, an increasing number of patients are diagnosed with AIS. However, there is no relevant research report on the incidence of postoperative VTE in these patients.

The incidence of postoperative VTE in patients with AIS may be significantly lower than in other adenocarcinoma types. Many factors can influence the occurrence of VTE, including patient-related risk factors (age, complications, previous VTE history, etc.), tumor-related risk factors (tumor diameter, nodule morphology, etc.) and treatment-related risk factors (chemotherapy, surgery, center Intravenous access, etc.) (19). In addition, previous studies have confirmed that different TNM stages significantly affect the occurrence of VTE events (20–22). Among them, Lee et al. (21) showed that the risk of VTE in patients with non-small-cell lung cancer increased 2.45 times with the progression of each stage. Cui et al. (22) also showed that patients with advanced stage disease were at increased risk of VTE. Previous studies have suggested that lung adenocarcinoma exhibits a natural progression pattern of AAH-AIS-MIA-IA with different degrees of malignancy (23–25). However, the influence of different degrees of malignancy of adenocarcinoma on the occurrence of VTE events is still unclear. In this study, the propensity score matching method (PSM) was used to eliminate the factors that interfered with the occurrence of VTE events between the two groups of patients with AIS and stage IA adenocarcinoma and confirmed for the first time that the incidence of postoperative VTE in patients with AIS was significantly lower than that in adenocarcinoma patients with other degrees of malignancy. Moreover, the author Ming S. Tsao of the recently released fifth edition of the World Health Organization (WHO) classification of thoracic tumors (13) considered AIS as a precursor gland lesion and removed the diagnosis of lung cancer to obtain a clearer tumor classification. On the other hand, as we all know, thymic tumors have a low grade of malignancy and a good prognosis. A previous study reported the incidence of VTE after thymectomy and evaluated the effectiveness of Caprini RAM (26), in which the incidence of VTE was 4.6% in patients with benign thymic disease and 14.5% in patients with malignant disease. The incidence of VTE in AIS that we reported was similar to the benign disease in the above study; Moreover, previous studies (27) have confirmed that AIS patients can be cured after complete resection, and the 10-year postoperative recurrence-free survival rate is almost 100%, which has verified the biological behavior of AIS as having low-grade malignant potential. Our study also confirmed this from the perspective of VTE, and patients with AIS have a lower risk of VTE after surgery, similar to the incidence of VTE after surgery for benign disease in thoracic surgery.

When applying the modified Caprini RAM to assess VTE risk in patients with adenocarcinoma in situ, it may be more appropriate not to score the “malignancy” item. Our study confirmed that when the “malignant tumor” item was not evaluated, the incidence of VTE in intermediate-high-risk patients was significantly increased from 7.9% to 36.4%. At this time, the modified Caprini RAM can effectively screen out the susceptible VTE population, with an excellent stratification effect. Whether the “malignancy” item is evaluated plays an important role in modified Caprini stratification in patients with AIS. The patients with AIS in this study were mainly younger female patients with fewer comorbidities, consistent with the baseline characteristics of patients in other studies (28–30), and these patients rarely have a long operation time. If the “malignancy” item remains, most postoperative patients are at intermediate risk (5-8 scores) in the modified Caprini risk stratification. The patients with AIS were further scored twice to retain and exclude the “malignant tumor” item, and then the incidence of VTE in patients at intermediate-high risk (≥5 scores) was compared between the two scores. The results showed that the “malignancy” item not being evaluated can effectively improve the accuracy of the modified Caprini RAM stratification. Therefore, in our study, it is believed that when Caprini RAM is applied to patients with AIS, excluding the “malignancy” item score will make the prediction accuracy higher.

The underlying mechanism of the lower risk of VTE in patients with AIS is unclear. However, previous studies (15, 16, 27, 31) have shown that adenocarcinoma *in situ* has a low-grade malignant potential with an almost 100% 5-year survival rate after surgical resection. Furthermore, studies (32, 33) have shown that the blood of patients with aggressive malignancies is hypercoagulable and prone to VTE. In this study, the preoperative and postoperative coagulation indicators of the two groups of patients after PSM were further analyzed. The results showed that the preoperative and postoperative D-dimer levels of patients with AIS were significantly lower than those of patients with stage IA adenocarcinoma. The biological behavior of AIS and malignant tumors may be different, and coagulation function is less affected; thus, patients with AIS have a significantly lower risk of VTE.

There are some limitations in this study. First, although PSM was applied to match as many variables as possible, there may still be potential confounding factors, which need to be further verified by prospective randomized controlled trials. Second, our study is a single-center study, which does not have universal applicability. Finally, monitoring for the occurrence of VTE was not continued after discharge, which may have underestimated the incidence of VTE.

## Conclusion

The incidence of VTE after surgery for AIS was significantly lower than that for patients with stage IA adenocarcinoma after surgery. When using the modified Caprini RAM to assess VTE risk in patients with AIS, higher predictive accuracy was achieved when the “malignancy” item was not evaluated. Our study confirmed that from the perspective of VTE, the diagnosis of adenocarcinoma *in situ* removed from lung cancer may be more appropriate.

## Data availability statement

The raw data supporting the conclusions of this article will be made available by the authors, without undue reservation.

## Ethics statement

The studies involving human participants were reviewed and approved by the Ethics Committee of Beijing Chaoyang Hospital Affiliated to Capital Medical University. Written informed consent for participation was not required for this study in accordance with the national legislation and the institutional requirements.

## Author contributions

Y-sC collected and analyzed the patient data and were the major contributors in writing this manuscript. H-hD and X-yL participated in data collection and collation. XY and SC contributed to methodology. J-bM and Q-rC contributed to designing and critically revising the article. BH and HL contributed to the article review. All authors read and approved the final manuscript.

## Acknowledgments

We want to thank all staff and patients enrolled in our study.

## Conflict of interest

The authors declare that the research was conducted in the absence of any commercial or financial relationships that could be construed as a potential conflict of interest.

## Publisher’s note

All claims expressed in this article are solely those of the authors and do not necessarily represent those of their affiliated organizations, or those of the publisher, the editors and the reviewers. Any product that may be evaluated in this article, or claim that may be made by its manufacturer, is not guaranteed or endorsed by the publisher.
