# Supplementary material for: Incidence of venous thromboembolism after surgery for adenocarcinoma in situ and the validity of the modified Caprini score: A propensity score-matched study
Source: Front Oncol. 2022 Sep 2;12:976988. doi: 10.3389/fonc.2022.976988 (PMC9478866; doi:10.3389/fonc.2022.976988)
Supplement: Supplementary file 1 [file Table_1.docx]

**Table Modified Caprini risk assessement model**

| **Caprini risk factor** | **Caprini score** |
| --- | --- |
| Age 40–59 (years) | 1 |
| Abnormal pulmonary function | 1 |
| Acute myocardial infarction (<1month) | 1 |
| BMI ≥30 (kg/m^2^) | 1 |
| congestive heart failure (<1 month) | 1 |
| history of inflammatory bowel disease | 1 |
| history of prior major surgery (<1 month) | 1 |
| Complications of pregnancy | 1 |
| Oral contraceptive use or HRT | 1 |
| Sepsis (<1 month) | 1 |
| Serious acute lung disease (<1 month) | 1 |
| Swollen legs (current) | 1 |
| Varicose veins | 1 |
| Age 60-74 (years) | 2 |
| Central venous access | 2 |
| Confined to bed (>72 hours) | 2 |
| Major open surgery (≥45 minutes) | 2 |
| Present cancer | 2 |
| Prior cancer, except nonmelanoma skin | 2 |
| Age ≥ 75 (years) | 3 |
| History of VTE | 3 |
| Family history of VTE | 3 |
| Chemotherapy | 3 |
| Positive anticardiolipin antibody | 3 |
| Positive Lupus anticoagulant | 3 |
| Acute spinal cord injury | 5 |
| Major surgery ≥6 hours | 5 |

BMI,body mass index;HRT,hormone replacement therapy; VTE,venous thromboembolism.
